# Effects of Long-Term In Vivo Exposure to Di-2-Ethylhexylphthalate on Thyroid Hormones and the TSH/TSHR Signaling Pathways in Wistar Rats

**DOI:** 10.3390/ijerph14010044

**Published:** 2017-01-04

**Authors:** Xinwen Dong, Jin Dong, Yue Zhao, Jipeng Guo, Zhanju Wang, Mingqi Liu, Yunbo Zhang, Xiaolin Na

**Affiliations:** Department of Environmental Hygiene, Public Health College, Harbin Medical University, Harbin 150081, China; dongxinwen118@yeah.net (X.D.); dongjin1992dj@sina.com (J.D.); zy244943459@163.com (Y.Z.); guojipeng90@163.com (J.G.); wangzhanju00@163.com (Z.W.); liumingqihlj@163.com (M.L.)

**Keywords:** DEHP, hypothalamus-pituitary-thyroid axis, TSH/TSHR, signaling pathways, molecular mechanisms, thyroid function

## Abstract

Di-(2-ethylhexyl)phthalate (DEHP) was a widely used chemical with human toxicity. Recent in vivo and in vitro studies suggested that DEHP-exposure may be associated with altered serum thyroid hormones (THs) levels, but the underlying molecular mechanisms were largely unknown. To explore the possible molecular mechanisms, 128 Wistar rats were dosed with DEHP by gavage at 0, 150, 300, and 600 mg/kg/day for 3 months (M) and 6 M, respectively. After exposure, expression of genes and proteins in the thyroid, pituitary, and hypothalamus tissues of rats were analyzed by Q-PCR and western blot, while the sera and urine samples were assayed by radioimmunoassay and ELISA. Results showed that serum THs levels were suppressed by DEHP on the whole. DEHP treatment influenced the levels of rats’ thyrotropin releasing hormone receptor (TRHr), Deiodinases 1 (D1), thyroid stimulating hormone beta (TSHβ), sodium iodide symporter (NIS), thyroid stimulating hormone receptor (TSHr), thyroperoxidase (TPO), thyroid transcription factor 1 (TTF-1), and thyroglobulin (TG) mRNA/protein expression in the hypothalamus-pituitary-thyroid (HPT) axis and decreased urine iodine. Taken together, observed findings indicate that DEHP could reduce thyroid hormones via disturbing the HPT axis, and the activated TSH/TSHR pathway is required to regulate thyroid function via altering TRHr, TSHβ, NIS, TSHr, TPO, TTF-1 and TG mRNA/protein expression of the HPT axis.

## 1. Introduction

Thyroid diseases are endocrine disorders of the body [1]. In addition to genetic factors, increasing evidence indicates that environmental factors, such as endocrine-disrupting chemical pollutants affect thyroid hormone levels, causing changes of thyroid morphology, autoimmune thyroid disease, and thyroid tumor, etc. [2,3,4,5]. Among these chemicals, the ubiquitous plasticizer di(2-ethylhexyl)phthalate (DEHP) has been reported to induce pathologic alterations in the thyroid in rats [6]. DEHP is often exposed to by the human bodies. After exposure, DEHP is easily metabolized, crosses the placenta, and is detected at higher concentrations in children than in adults [7].

DEHP is considered as antiandrogenic endocrine disruptors because of its possible effect on animal gonads and reproduction [8,9]. A growing number of research has been focused on its role in the disruption of the reproductive and developmental processes [7]. On the other hand, very few studies focused on the thyroid-disrupting effects of DEHP [10,11]. A recent in vitro study reported that DEHP and other phthalates caused changes in the iodide uptake of thyroid follicular cells [12]. In animal studies, rats fed with diets contaminated with DEHP were found to have thyroid alterations and lower plasma thyroxine (T4) concentrations compared with controls [13,14,15,16]. Additionally, rodent studies found that histopathological changes in the rats’ thyroid after exposure to DEHP corresponding to hyperactivity of the thyroid [13,14,15,16,17].

A cross-sectional relationship study in the United States revealed that DEHP metabolites, total T4 (TT4), free T4 (FT4), and total triiodothyronine (TT3) were negatively correlated with thyroglobulin (TG), and were positively correlated with thyroid stimulating hormone (TSH) [18]. This suggested that DEHP exposure was related to thyroid hormone synthesis, release, transport, and metabolism, possibly by impeding the role of the hypothalamus or pituitary. In adolescents, DEHP metabolites and TT3 were significantly positively correlated [18]. A cross-sectional study with Danish children found that DEHP metabolites were negatively correlated with TT3 and free triiodothyronine (FT3) levels in urine [19]. Wu et al. conducted a study on food contamination events in Taiwan in 2011 and found that serum thyroid stimulating hormone levels and histopathology of thyroid glands were changed after consumption of food contaminated with high concentrations of phthalates [20]. These studies strongly linked DEHP to thyroid diseases in human.

Very few studies has been conducted on the thyroid-disrupting effects of DEHP, leaving underlying mechanisms largely unknown. Therefore, the aim of this study is to investigate the effects of DEHP on the hypothalamic-pituitary-thyroid (HPT) axis and explore the potential mechanisms for this disruption.

## 2. Materials and Methods

### 2.1. Chemicals and Reagents

The products used in this study were DEHP (Tokyo Chemical Industry Co., Ltd., Tokyo, Japan). Distilled water was filtered using a Milli-Q system (Millipore, Billerica, MA, USA). The DEHP standard (purity 99.6%) was purchased from the National Institute of Standards and Technology (Gaithersburg, MD, USA). Potassium iodate (Aladdin, Shanghai, China). Urine iodine standard reference materials (GBW09109h, National Institute of Standard Substance, Beijing, China). All other chemicals, reagents, and buffers were analytical-grade products from Amresco LLC. (Solon, OH, USA). 

### 2.2. Animals and Treatments

One hundred and twenty-eight healthy female Wistar rats aged 4–6 weeks and weighing 60–80 g were obtained from Vital River Laboratory Animal Technology Co. Ltd. (Beijing, China). All animal experimentation was performed in accordance with the Institute of Zoology Animal and Medical Ethics Committee of Harbin Medical University (Grant No. 2015010) and was in accordance with the current Chinese legislation. The rats were housed and adapted to new surroundings for 7 days under a controlled temperature ranging between 20 and 24 °C, at a controlled humidity of 50%–60%, with a 12-h light/dark cycle. The rats were provided with standard AIN-93M diets and housed individually in stainless steel, wire-mesh cages. 

These animals were randomly assigned into 4 groups (n = 32/dose group): the low-dose group, gavage at a dose of 150 mg/kg/day (about five times the “No Observed Adverse Effect Level”, NOAEL); the middle-dose group, gavage at a dose of 300 mg/kg/day (about ten times the NOAEL); the high-dose group, gavage at a dose of 600 mg/kg/day (about twenty times the NOAEL); and the control group, gavage at a dose of 0 mg/kg/day. The NOAEL was obtained from a 104-week study in rats, which was from the 2000 chronic toxicity assessment of di(2-ethylhexyl)phthalate in rats [21]. The dosage design was selected to establish a dose range that would not be lethal, but could cause serum hormone changes and allow other possible target organ effects. The dose of 600 mg/kg is known to be able to induce adverse effects in rats without causing systemic toxicity in the short term [22]. DEHP was administered to rats continually for 24 weeks by gavage. Diet and drinking water was supplied as routine throughout the study. Rats were observed twice daily, and clinical findings were recorded. Animals were dosed daily by gavage for six months with DEHP (purity 99.9 %), which was dissolved in corn oil at 0 (control), 150, 300, and 600 mg/kg body weight. The age of animals and duration of treatment were based on the recommendations of the U.S. EPA Endocrine Disrupter Screening and Testing Advisory Committee (EDSTAC). 

### 2.3. Sample Collection and Preparation

All rats’ body weights were measured at the end of each week. Half of all animals in each group randomly were sacrificed under sodium pentobarbital anesthesia at 3 M and 6 M. Urine samples of each rat were collected in metabolic cages for 24 h over ice packs at 3 M and 6 M. Then centrifuged (10,000 rpm, 10 min), and the supernatants were collected and stored at −80 °C until analysis. The blood samples were obtained from the aorta abdominal before the rats were sacrificed. The blood samples were then allowed to clot. Serum was obtained by centrifugation at 3000 rpm for 15 min and then stored at −80 °C until hormonal analysis. Tissues of thyroid, liver, brain and pituitary samples was rapidly frozen in liquid nitrogen and stored at −80 °C until use. 

### 2.4. Iodine Level Detection

The levels of urine iodine were determined using UV-visible spectrometry according to a national standardized method in China. Briefly, urine or serum samples were digested by heating together with ammonium persulfate and chlorate. After digestion, arsenic acid and ammonium ceric sulfate was added to samples. Potassium iodate was used as iodine standard, obtained with standard reference materials (GBW09109h). The iodine levels in diluted samples and standard samples were determined by a double-beam UV-Vis spectrophotometer (TU-1901, Beijing Puxi Instruments, Beijing, China).

### 2.5. Haematoxilin-Eosin (HE) Staining and Histological Evaluation

Thyroids and livers were fixed in 10% formalin, processed and trimmed, embedded in wax blocks, sectioned to a thickness of 4 μm, and stained with hematoxylin and eosin (H&E) and then examined by light microscopy. 

### 2.6. Radioimmunoassay RIA

Serum TT4, TT3, FT4, FT3 and TSH levels were measured using radioimmunoassay kits (Beijing North Institute of Biological Technology, Beijing, China) according to the instruction guide strictly, by an Automatic Gamma Counter (Wallac Wizard-2^®^ 2470, Perkin Elmer, Wellesly, MA, USA). 

### 2.7. Enzyme-Linked Immunoabsorbent Assay

Serum thyrotropin releasing hormone (TRH) and thyroid stimulating antibody (TSAb) levels were measured using Elisa kits (Shanghai Guduo Institute of Biological Technology, Shanghai, China) according to the instruction guide strictly. All samples and standards were set repeatedly and detected on a BioTek instrument (Cytation 3 MFD, Bojue Co., Winooski, VT, USA), and using the standard curve for data analysis. No special cross-reactivity or interference was found in this test.

### 2.8. RNA Extraction and Real-Time Quantitative PCR

Total RNA in the rats’ thyroid tissues, which were intervened by DEHP for 0, 150, 300, 600 mg/kg/bw, was extracted with trizol reagents (Invitrogen, Carlsbad, CA, USA) and frozen mix by ball mill MM400 (Retsch GmbH, Haan, Germany). cDNA was synthesized from 1 μg total RNA (65 °C for 5 min and rapid cooling on the ice) using ReverTra Ace qPCR RT kit (Toyobo Co., Ltd., Life Science Department, Osaka, Japan). Reverse transcription (37 °C for 15 min and 98 °C for 5 min). qRT-PCR were performed by using THUNDERBIRD SYBR^®^ qPCR Mix with 50 × ROX reference dye (Toyobo Co., Ltd., Life Science Department) according to the manufacturer’s instructions. Thermo cycling was run on ABI-7500 PCR Detector System (Applied Biosystems, Foster, CA, USA) with the following temperature cycles: Initial denaturation at 95 °C for 1 min, followed by 40 cycles of 15 s denaturation at 95 °C, and 1 min elongation at 60 °C. The dissociation curve analysis of specificity of amplified products was performing at the end of each amplification and finally a cooling step of 30 °C. All the amplicons generated a single peak, thus reflecting the specificity of the primers. Expression of each target gene (thyroid stimulating hormone beta, TSHβ; sodium iodide symporter, NIS; stimulating hormone receptor, TSHr; thyroid transcription factor 1, TTF-1; thyroperoxidase, TPO; thyroglobulin, TG; Deiodinases 1, D1; thyrotropin releasing hormone receptor, TRHr) were measured in duplicate, and determined simultaneously for all samples in a 96-well plate. The mean of the reference normalized expression measurements (ΔCt) for duplicates was used for statistical analysis. The relative expression of target gene was calculated using 2^−ΔΔCt^, which is a relative quantitative calculation method applied in the detection of gene expression. The primer sequences were designed according to the cDNA sequences from the GenBank (Table 1). All primers were synthesized by the Invitrogen Custom Primers (Shanghai, China). 

### 2.9. Western Blot Analysis

Thyroids of each rat were fully ground and mixed in 600-μL cell lysis buffer for western blot and immunoprecipitation (IP) (Beyotime Biotech Inc., Shanghai, China) with 2 μL PMSF (100 mM). The protein concentrations were measured by the BCA Protein Assay Kit (Beyotime Biotech Inc.). Equal amounts protein in each sample was mixed with loading buffer, which was heated at 95 °C for 5 min before electrophoresis in 10% SDS polyacrylamide gel, and transferred to a polyvinylidene difluoride membranes. Membranes were incubated with 10% skim milk for 1 h, and incubated overnight at 4 °C with NIS, TSHr, TTF-1, TG polyclonal antibody (Santa Cruz Technology Inc., Santa Cruz, CA, USA, dilution: 1:800, 1:200, 1:500, 1:200, 1:500 respectively), TPO polyclonal antibody (Abcam, Cambridge, MA, USA, dilution: 1:200), TSHβ monoclonal antibody (R&D Systems Inc., Minneapolis, MN, USA, dilution: 1:200), and anti-GAPDH (Santa Cruz, dilution: 1:500). After rinsing three times (10 min each) with 1% Tris Buffered Saline with Tween, membranes were incubated with an AP-conjugated secondary antibody shown as Table 2 for 1 h at 37 °C, and rinsing again similarly. The Investigator ProImage software was used to visualize the specific protein. The relative expression of target protein was calculated using Quantity One.

### 2.10. Statistical Analysis

Quantitative results were expressed as mean ± standard deviation (SD). Significance was assessed by one-way analysis of variance (ANOVA) following appropriate transformation to normalized data and equalized variance where necessary. Mean values were compared by least-significant difference (LSD) using the SPSS statistical package 17.0 (SPSS Inc., Chicago, IL, USA). 

The statistical difference between control and DEHP-treated group was compared by Student’s *t*-test. The comparison among different groups were conducted by one-way analysis of variance (ANOVA) with a post-hoc test (Dunnet’s test), using GraphPad Prism Version 5 software (GraphPad Software Inc., San Diego, CA, USA). The statistical was set at *p* < 0.05 or *p* < 0.01.

## 3. Results

### 3.1. Body Weight (BW)

**Figure 1:**
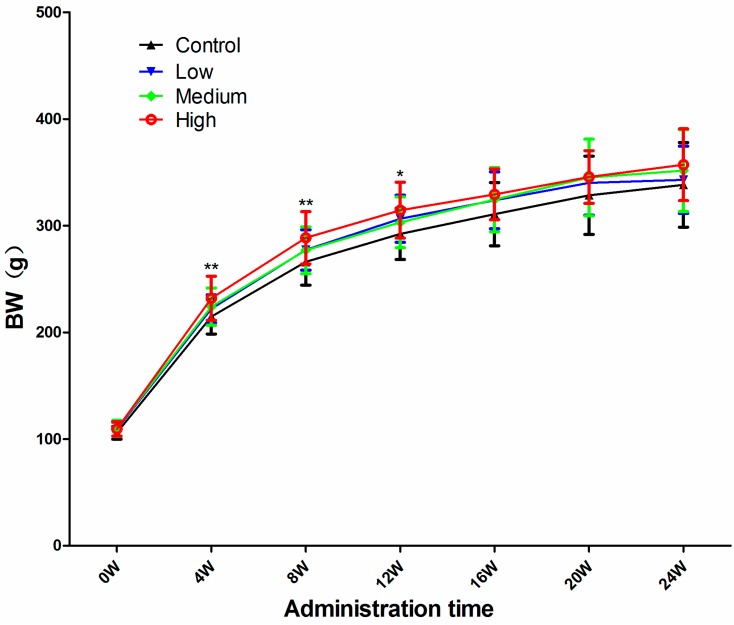
Body weight (BW) of rats at each time point and dose. Data are presented as means ± standard deviation (SD). * Significantly different from the control (*p* < 0.05); ** Significantly different from the control (*p* < 0.01).

The BW of all DEHP-treated rats were significantly higher during the entire treatment period, starting from the first week, and gradually stabilized after 12 weeks (Figure 1). This suggest that the body weights of the rats should be sensitive to the toxicity induced by long-term exposure to DEHP.

### 3.2. DEHP Elevated Relative Thyroid Weights and Reduced Urine Iodine Levels of Treated Rats

In this study, the levels of iodine in urine were significantly lower in all DEHP-treated groups compared with the control group at 3 M and 6 M (*p* < 0.05), and the levels of iodine in urine were significantly lower in high DEHP-dosed group compared with the medium- and low- dosed groups at 3 M and 6 M (*p* < 0.05) (Table 3). Relative thyroid weights were significantly increased only in the high-dose group compared with the control at 3 M (*p* < 0.05), and there was a upward trend in all DEHP-treated groups compared with the control at 6 M (*p* > 0.05) (Table 3).

### 3.3. Histological Changes in the Thyroid and Liver

**Figure 2:**
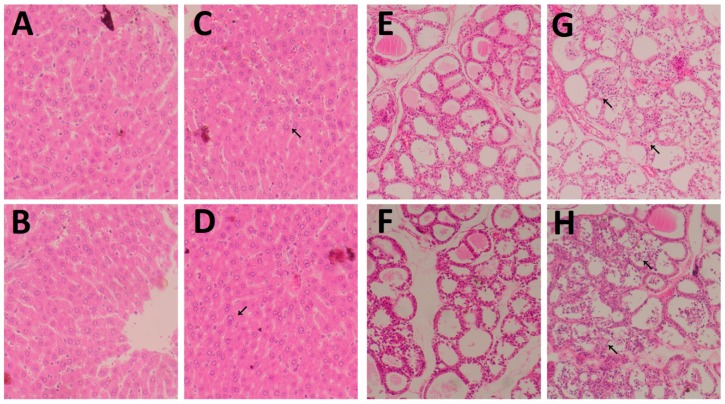
Effects of DEHP on the histology of thyroids and liver. Histopathology of thyroid and liver sections collected from Wistar rats treated with DEHP by gavage for 3 M and 6 M. Representative photomicrographs of H&E-stained formalin-fixed thyroid and liver sections from control group or high DEHP-dosed group. Histopathology of liver: (**A**) Control group at 3 M; (**B**) Control group at 6 M; (**C**) High-dose group at 3 M; (**D**) High-dose group at 6 M. Histopathology of thyroid: (**E**) Control group at 3 M; (**F**) Control group at 6 M; (**G**) High-dose group at 3 M; (**H**) High-dose group at 6 M. Control group (0 mg/kg/day); High-dose group (600 mg/kg/day). Arrows indicate hypertrophy and hyperplasia of follicular epithelial cells. Magnification: ×400 (liver) and ×200 (thyriod).

Histopathological changes in the livers and thyroids were observed in the high-dose group, in the 3 M and 6 M, respectively. Histopathological changes were characterized by pronounced fat degeneration, fat drops, empty lipocytes, and apoptosis cells in the high-dose group, compared with the control group at 3 M and 6 M after exposure to DEHP (Figure 2A–D). 

Fatty degeneration and vacuolar degeneration in the liver cytoplasm were present in the high-dose group after 3 M (Figure 2C). Fatty degeneration, vacuolar degeneration in the cytoplasm of hepatocytes, and hepatocyte necrosis were present in the high-dose group after 6 M of dosing (Figure 2D). On the other hand, multiple alterations in the thyroid were observed in the DEHP-treated groups: parts of the follicular cavity were increased, and filled with a lot of dyed gelatin (Figure 2E–H). In the high-dose group, follicular hyperplasia is more obvious (Figure 2G,H). In the thyroid of these rats, thyroid follicular was unequal in size, while the follicular cavity was filled with colloid (Figure 2G). Also, more follicular epithelium was found shed in the hair cavity of the follicular cavity (Figure 2G). Follicular cavity was smaller, and more follicular epithelium was found fell off (Figure 2H).

### 3.4. DEHP Altered Serum Levels of Thyroid Hormones

**Figure 3:**
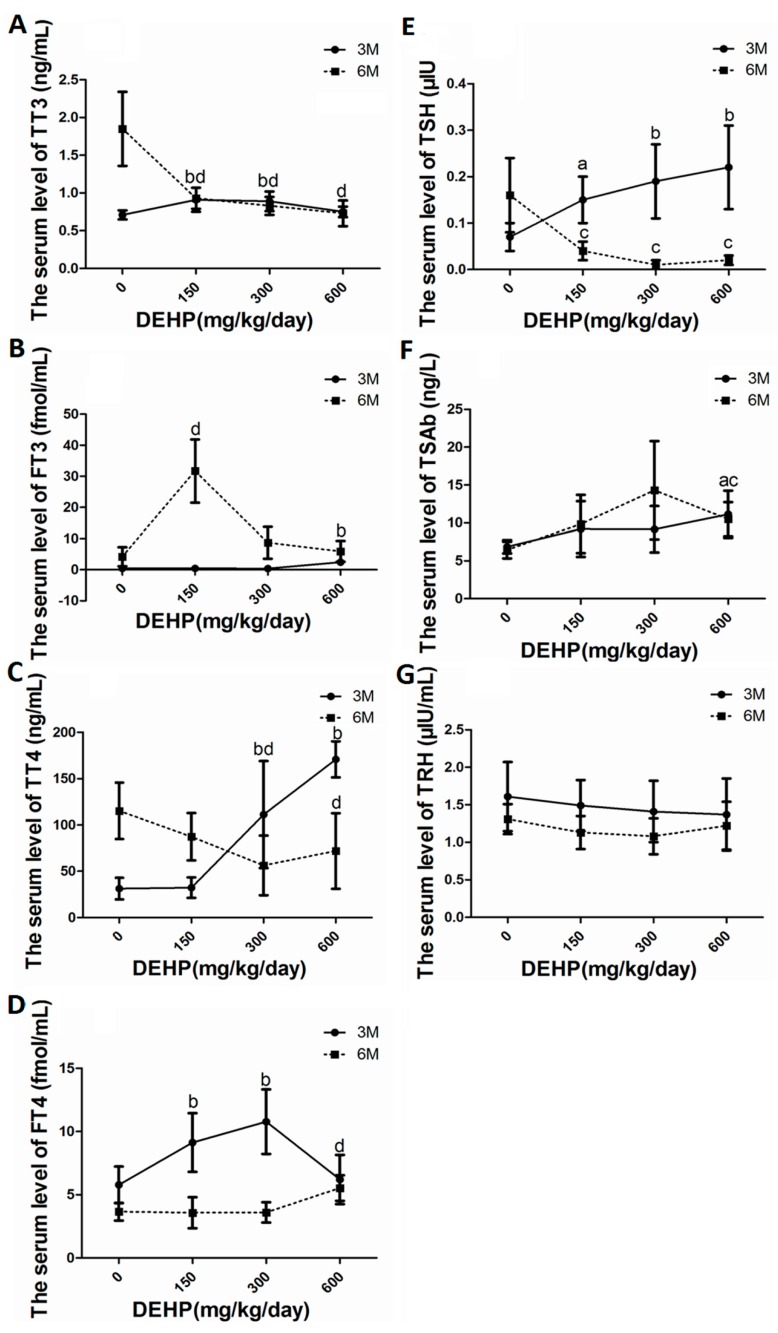
Effects of long-term exposure to DEHP on the serum levels of thyroid-related hormones at 3 M and 6 M respectively. Groups of different doses of DEHP: 0 mg/kg/day, control group; 150 mg/kg/day, low-dose group; 300 mg/kg/day, middle-dose group; 600 mg/kg/day, high-dose group. Each bar represents the mean ± S.E.M (16/group). ^a^ Significantly different from the control at 3 M (*p* < 0.05); ^b^ Significantly different from the control at 3 M (*p* < 0.01); ^c^ Significantly different from the control at 6 M (*p* < 0.05); ^d^ Significantly different from the control at 6 M (*p* < 0.01). (**A**) TT3; (**B**) FT3; (**C**) TT4; (**D**) FT4; (**E**) TSH; (**F**) TSAb; (**G**) TRH. Radioimmunoassay: (**A**–**E**); Enzyme-linked immunoabsorbent assay: (**F**,**G**).

After exposure to DEHP, THs in serum demonstrated a decreasing trend at 3 M and an increasing trend at 6 M on the whole. In this study, TT3, FT3, TT4, FT4, TSH, and TSAb in groups of different DEHP doses were all increased compared with the control at 3 M (*p* < 0.05) (Figure 3A–E). Likewise, FT3, FT4 in the DEHP-dosed group were lower than those in the control (*p* < 0.05). However, with the increase of DEHP-treated dose, rats serum TT3, TT4 and TSH level in all groups of different DEHP doses were lower than the control group at 6 M (*p* < 0.05). Moreover, Elisa detection results indicated that DEHP treatment increased serum TSAb levels (Figure 3F) and decreased serum TRH levels (Figure 3G) when compared with the control group at 3 M and 6 M (*p* < 0.05).

### 3.5. DEHP Influenced mRNA Expressions of Hypothalamus TRHr, Pituitary TSHβ, Thyroid TPO, TSHr, NIS, Tg, TTf-1, D1 and Liver Deiodinase 1

**Figure 4:**
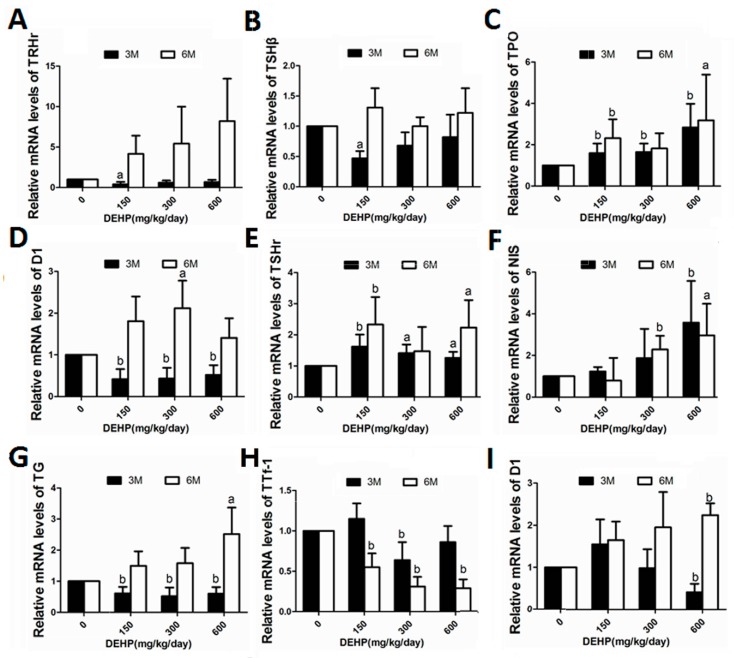
Effects of long-term exposure to DEHP on the mRNA expression levels of TRHr, TSHβ, TPO, D1, TSHr, NIS, TG and TTf-1 at 3 M and 6 M respectively. All rats were treated with DEHP (0, 150, 300, 600 mg/kg/day) by gavage for 3 M and 6 M, and the effect of this treatment on the expression of above-mentioned thyroid-related genes were examined by QPCR. Groups of different doses of DEHP: 0 mg/kg/day, control group; 150 mg/kg/day, low-dose group; 300 mg/kg/day, middle-dose group; 600 mg/kg/day, high-dose group. Data are presented as the mean ± S.E.M (6/group). ^a^ Significantly different from the control (*p* < 0.05); ^b^ Significantly different from the control (*p* < 0.01). (**A**) TRHr; (**B**) TSHβ; (**C**) TPO; (**D**) D1; (**E**) TSHr; (**F**) NIS; (**G**) TG; (**H**) TTf-1; (**I**) D1. Hypothalamus: (**A**); Pituitary: (**B**); Thyroid: (**C**–**H**); Liver: (**I**).

DEHP exposure resulted in time-dependent differential alterations in the genes expression. Exposure to DEHP at low dose for 3 M led to signicant lower mRNA levels of hypothalamus TRHr (Figure 4A). When the exposure extended to 6 M, however, such expression seemed to increase in a dose-dependent manner (Figure 4A). 

Low dose of DEHP treatment for 3 M led to significant lower gene expression of pituitary TSHβ (Figure 4B). However, only modest changes in expression was found after 6 M exposure to DEHP for such gene (Figure 4B). 

After exposure to DEHP for 3 M, gene expression of deiodinase1 in the rat liver showed an upward trend in the low-dose group, but was significantly reduced in the high-dose group (Figure 4I). When the exposure to DEHP extended to 6 M, however, the expression of deiodinase1 in the liver exhibited a dose-dependent increase, and reached significance in the high-dose group (Figure 4I).

For thyroid genes expression, 6 M treatment of DEHP led to overall increase in genes expression, such as TPO (Figure 4C), thyroid D1 (Figure 4D), thyroid TSHr (Figure 4E), NIS (Figure 4F), and TG (Figure 4G), except that TTf-1 expression was found signficantly lower at 6 M after DEHP exposure (Figure 4H). Whereas for 3 M treatment of DEHP, significantly lower D1 and TG was detected (Figure 4D,G).

### 3.6. DEHP Influenced Protein Expressions of NIS, TSHr, TPO, TG, TTf-1 and TSHβ

**Figure 5:**
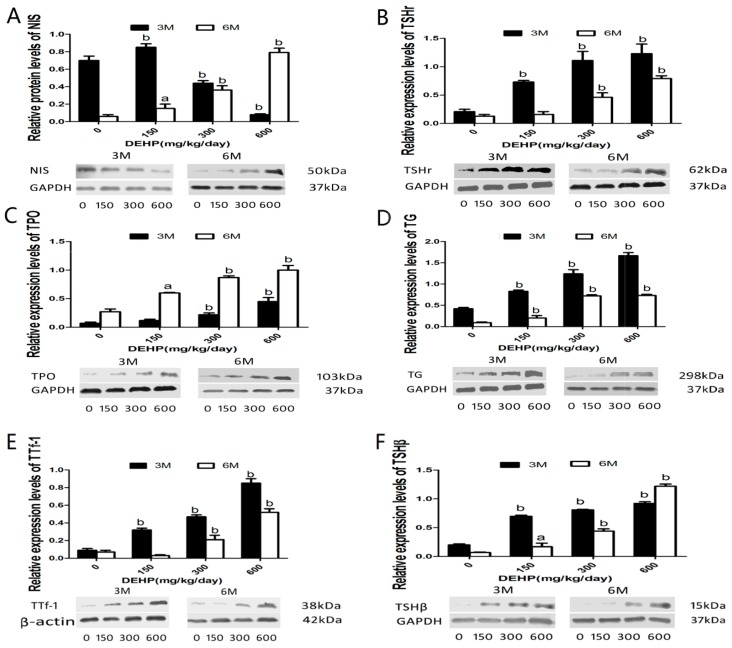
Effects of long-term exposure to DEHP on the protein expression levels of NIS, TSHr, TPO, TG, TTf-1 and TSHβ at three and six months respectively. All rats were treated with DEHP (0, 150, 300, 600 mg/kg/day) by gavage for 3 M and 6 M, and the effect of this treatment on the expression of above-mentioned thyroid and Pituitary related proteins were examined by western blot. Groups of different doses of DEHP: 0 mg/kg/day, control group; 150 mg/kg/day, low-dose group; 300 mg/kg/day, middle-dose group; 600 mg/kg/day, high-dose group. Data are presented as the mean ± S.E.M (6/group). ^a^ Significantly different from the control (*p* < 0.05); ^b^ Significantly different from the control (*p* < 0.01). (**A**) NIS; (**B**) TSHr; (**C**) TPO; (**D**) TG; (**E**) TTf-1; (**F**) TSHβ. Thyroid: (**A**–**E**); Pituitary: (F).

To detect effects of DEHP on the biotransport of THs, protein levels of TSHβ, TTf-1, TSHr, TG, TPO, and NIS were analyzed. Protein levels of thyroid NIS in both middle and high-dose groups, but not in the low-dose group, were significantly decreased at 3 M (*p* < 0.01), while significant increases were observed in groups of different doses of DEHP at 6 M in comparison with the control (*p* < 0.05). On the other hand, the levels of thyroid TSHr, TPO, TG, TTf-1, and pituitary TSHβ protein expression were increased in all DEHP-treated groups at both 3 M and 6 M (*p* < 0.05) (Figure 5).

## 4. Discussion

Strong evidence from previous human and animal studies suggested that DEHP has many adverse effects on the thyroid function [6,18,20,23]. However, the underlying molecular mechanisms were not fully understood. In order to observe the long-term effects on THs, rats were exposed to different doses of DEHP for consecutive 24 weeks. The results showed that serum THs levels were dysregulated after DEHP exposure. Genes and protein expression in the HPT axis were altered, while urine iodine was decreased. Taken together, observed findings indicate alterations of TSH/TSHR signaling pathways were the key molecular mechanisms underlying the impact of DEHP on female rats’ thyroid in vivo. 

Urinary iodine is an evaluation index of iodine intake, which is closely related with thyroid function [24]. In this research, the results suggested the relative thyroid weights were increased, and whole serum THs and urine iodine levels were decreased. In addition, thyroid morphology has been altered after long-term exposure to DEHP. Moreover, DEHP-treatment had an effect on rats’ growth. All the results indicated that long-term and excessive consumption of DEHP can break the self-regulatory function of thyroid. Increasing doses of DEHP activated the HPT axis accompanied by the emergence of hypothyroidism, which appears to stimulate the thyroid follicular hyperplasia. These results are consistent with Poon’s research about rats fed with DEHP for 90 days [15]. However, the mechanism of thyroid toxicity of DEHP is currently unclear.

Most often, two regulatory pathways existed in thyroid when facing the external environment stimulation. Firstly, the autoregulatory mechanism of the thyroid is started when the thyroid function is affected. In the basolateral membrane, iodine intake was mediated by NIS from plasma against a concentration gradient [25]. Upon transportation across the epithelial cell, iodine was catalyzed by TPO in the neighboring follicular cavity at the top of the cell membrane. After activation, iodine and Tg molecules formed mono- and diiodotyrosines (MIT and DIT). Two DIT molecular couplings give T4, or a molecule of MIT condensed with a molecule of DIT to give T3, and stored in the follicular cavity gum in the form of combination with Tg. Upon thyroid hormone secretion, follicular epithelial cells transported Tg from the follicular cavity to the epithelial cells through micropinocytosis, released T3, T4 and TSH under the action of lysosomal proteolytic enzyme, and secreted into the bloodstream at the basolateral membrane in a certain proportion under the action of thyroid D1 [26].

In our experiment, we found that DEHP intervention increased NIS gene and protein expression levels in thyroid after 6 M, which resulted in increased uptake of iodine from plasma, as well as decreased urine iodine level. Since iodine needs TPO activation, and therefore the altered iodine levels resulted in an upward trend of TPO mRNA and protein in this experiment at 6 M, which was further combined into T3, T4 after iodide organification with TG molecules. Therefore, TG gene and protein expression after 6 M were both increased. Meanwhile, our research also indicated that DEHP intervention resulted in a decreased serum THs level at 6 M, suggesting that DEHP intervention in this experiment can affect the endocrine system, followed by the increased THs secretion of the pituitary hormones ensure a relatively stable thyroid hormone levels in the body cycle. Thus, affecting TH biotransport could be considered as a reason of the reduction of TH levels induced by DEHP. 

In the second regulatory pathway, a further over-stimulation will result in an activated HPT axis. The hypothalamus controls hormone TRH secretion from the nearby pituitary gland and can promote the synthesis and secretion of pituitary TSH. TSHβ secreted from pituitary gland is the main hormone that promotes the synthesis and secretion of thyroid hormones. TSAb could conjugate with TSH receptor through the adenylate cyclase-cAMP way to produce a similar TSH biological effect, further inducing an increased level of cAMP [27]. TSH-TSHR-cAMP-PKA signaling pathway is critical for thyroid function, which represents a positive regulatory pathway. Extracellular signal is incorporated with thyroid TSHR on the cell membrane combined into intracellular signals. TSHr is activated by Gs protein, which can change adenylate cyclase activity and catalysis ATP converted into cyclization adenosine (cyclic adenylic acid, cAMP). Once the concentration of intracellular cAMP changed, the adjustment role of cAMP on cells is by activating the protein kinase A (protein kinase, PKA) system to realize the cascade reaction of cAMP that will eventually enhance gene expressions of thyroid specific transcription factors, such as TTf-1 gene expression and activity, thus improving the thyroid cell NIS mRNA and protein expression [28,29,30]. As a result, the thyroid iodine intake function is enhanced, possibly leading to decreased serum THs and urine iodine. 

In this study, we found that DEHP induced a downward trend in the hypothalamus TRHr mRNA and serum TRH after 6 M. At the same time, the results of our study showed that elevated expression levels of pituitary TSHβ gene and protein were induced by DEHP. The above findings indicated that the normal feedback regulation of HPT axis was disrupted after exposure to DEHP. Elevated levels of TSHr gene and protein expression in our study suggested that cAMP activity could be enhanced by DEHP. Since it has been confirmed that TSHr transcriptional regulation is mainly by the TTf-1 route via the ATP-cAMP-PKA pathway [31], we believe that high expression of TSHr protein may result from the increase of TTf-1 protein expression and activity. Our results also demonstrated that DEHP intervention could prompt the upregulation of TTf-1 and TSHr and cause an increase in TSH/TSHR signaling, which can increase the expression of thyroid tissue NIS and the secretion of thyroid hormones. This negative feedback regulation is of great significance in maintaining a certain degree of signal strength, avoiding over-stimulation and maintaining functional stability, so we concluded that DEHP has an influence on follicular cavity iodide TG by regulating TTf-1, and further upregulated TSHr expression, then changed the follicle sensitivity to central TSH/TSHR signaling. The aforementioned results also indicated a feedback suppression on TRH and TSH production via the negative feedback system of the HPT axis. Eventually, decreased TRH eliminated the production of T3 and T4 in the thyroid. Ghiscari [10] has shown that, DEHP can promote T3-dependent GH3 cell proliferation, while inhibiting the GH3 cell proliferation under the action of T3.

Moreover, another mechanism found in this study was related to liver function disorder, which could be considered as a reason for THs alterations. D1 is a TH-activating enzyme that catalyze the conversion of the prohormone T4 to the active hormone T3. Research has shown that [32], iodine deficiency led to increased D1 activity of the thyroid tissue and increased T4 to T3 conversion. In the present study, the activity of D1 in the thyroid was enhanced by DEHP, which may be a possible reason for increase in the conversion of T4 to T3. A similar change of D1 was also found in the liver in our study, which suggested that thyroid dysfunction was accompanied with the liver metabolism disorder after long-term exposure to DEHP. In addition, increased relative liver weights and the liver pathological alterations appeared in our experiments, which could further prove the aforementioned results. Since T3 is the major active form of thyroid hormone [33], the main role of these changes is to maintain the supplementation of serum T3 levels, to protect the need of the body’s metabolism.

## 5. Conclusions

In a word, the substantial morbidity and mortality of thyroid diseases in recent years all over the world calls for attention to the influences of phthalic acid ester materials on thyroid function. To address this issue, a dose and time dependent study of in vivo exposure to the phthalate DEHP was carried out. Our results showed that 6-month in vivo DEHP exposure influenced thyroid hormone levels, and further causes imbalance of the HPT axis of the body through TSH/TSHR signaling, which could be considered as an important mechanism of thyroid toxicity caused by DEHP. DEHP played a vital role in decreasing levels of serum THs, while resulting in differential alterations in genes and proteins expression in the hypothalamus, pituitary, and thyroid. These findings will help to reveal the real cause of thyroid disease and its relationship with DEHP. 

## Figures and Tables

**Table 1:** Description of primers used in the present study.

Primer	Type	Primer Sequence	GenBank
TSHβ	Forward	5‘-TACTGCCTGACCATCAACACC-3’	NM_013116.2
	Reverse	5’-GGTAGGAGAAATAAGGAGCAACAT-3’	
NIS	Forward	5’-CAGTTCTGGAATGGACACGG-3’	NM_052983.2
	Reverse	5’-TCTTGGTCACAGCAGGGATG-3’	
TSHr	Forward	5’-GTGGGAATAAGCAGCTACGC-3’	NM_012888.1
	Reverse	5’-GGATTTCGGACGGTGATGT-3’	
TTF-1	Forward	5’-GCACTTGGAGTAAGGCAGAAA-3’	XM_006224320.2
	Reverse	5’-ACCCCACGATACACGAACC-3’	
TPO	Forward	5’-ATGAGGCTGTGACTGAAGATGA-3’	NM_019353.2
	Reverse	5’-GTGGTCCGTGAGGAGTTTGA-3’	
TG	Forward	5’-GCCCTAACTCATCCGTCCA-3’	NM_030988.2
	Reverse	5’-TGTTGATAAGCCCATCGTCCT-3’	
D1	Forward	5’-GCAGACCCCTGGTGTTGAA-3’	NM_021653.3
	Reverse	5’-GGTCCTGGAGGCTTCGGT-3’	
TRHr	Forward	5’-AGGAGTCAGACCGCTTTAGCA-3’	NM_013047.3
	Reverse	5’-GAACTGGGTCCATTCTTCTCG-3’	
β-actin	Forward	5’-CCGTAAAGACCTCTATGCCAACA-3’	NM_013116.2
	Reverse	5’-GGGGCCGGACTCATCGTA-3’	

**Table 2:** Primary and Secondary antibodies used for western blot.

Primary Antibodies	Primary Source	Dilution	Brand	Second Source	Dilution
GAPDH	Rabbit	1:800	Santa Cruz, USA	Anti-Rabbit	1:800
NIS	Goat	1:800	Santa Cruz, USA	Anti-Goat	1:800
TSHr	Goat	1:200	Santa Cruz, USA	Anti-Goat	1:800
TTf-1	Rabbit	1:500	Santa Cruz, USA	Anti-Rabbit	1:800
TPO	Rabbit	1:200	Abcam, USA	Anti-Rabbit	1:800
TG	Goat	1:500	Santa Cruz, USA	Anti-Goat	1:800
TSHβ	Goat	1:250	R&D Systems, USA	Anti-Mouse	1:800

**Table 3:** Tissue weights and urine iodine levels of rats in groups of different doses at 3 M and 6 M.

Group	Liver/Weight (g/100 g)		Thyroid/Weight (g/100 g)		Urine Iodine (μg/L)	
	3 M	6 M	3 M	6 M	3 M	6 M
C	2.29 ± 0.73	2.46 ± 0.17	8.19 ± 1.61	8.27 ± 2.63	160.82 ± 43.56	95.73 ± 14.47
L	2.69 ± 0.32 ^a^	2.55 ± 0.34	8.51 ± 1.46	10.48 ± 2.85	147.90 ± 31.79	70.92 ± 17.27 ^b^
M	2.79 ± 0.17 ^b^	2.71 ± 0.25 ^a^	8.55 ± 1.76	10.59 ± 2.00	133.36 ± 24.58	72.29 ± 18.97 ^b^
H	3.78 ± 0.46 ^b^	3.00 ± 0.30 ^b^	10.87 ± 2.63 ^b^	12.50 ± 6.65	79.04 ± 15.46 ^b^	71.24 ± 13.90 ^b^

Values expressed as mean ± SD. C, control group (0 mg/kg/day); L, low-dose group (150 mg/kg/day); M, middle-dose group (300 mg/kg/day); H, high-dose group (600 mg/kg/day). ^a^ Significantly different from control (0 mg/kg/day) rats at *p* < 0.05. ^b^ Significantly different from control (0 mg/kg/day) rats at *p* < 0.01 (One-way ANOVA).
